# The effect of family health on suboptimal health status: The parallel mediation role of sleep quality and health behaviour

**DOI:** 10.7189/jogh.14.04071

**Published:** 2024-04-05

**Authors:** Lai Kun Tong, Mio Leng Au, Mu Rui Zheng, Yong Bing Liu, Guang Lei Fu, Yue Yi Li

**Affiliations:** 1 Research Management and Development Department, Kiang Wu Nursing College of Macau, Macao, China; 2 Education Department, Kiang Wu Nursing College of Macau, Macao, China; 3 Faculty of Health Sciences, University of Macau, Macao, China; 4 School of Nursing, Yangzhou University, Yangzhou, China; 5 Infectious Disease Department, The First Affiliated Hospital of Jinan University, Guangzhou, China

## Abstract

**Background:**

The prevalence of suboptimal health status has been increasing worldwide, posing a significant challenge to public health. Meanwhile, family health has been recognised as an important factor influencing individual health outcomes. However, the mechanisms through which family health affects suboptimal health status remain unclear. We aimed to investigate the parallel mediation role of sleep quality and health behaviour in the relationship between family health and suboptimal health status.

**Methods:**

We conducted a cross-sectional online survey with a sample of adults >18 years old from four provinces in China. The survey questionnaires queried their demographic characteristics, family health, suboptimal health status, sleep quality, and health behaviour. We assessed family health by the Family Health Scale-Short Form and suboptimal health status using the Suboptimal Health Status Questionnaire. We employed structural equation modelling to analyse the data and test the proposed mediation model.

**Results:**

we collected 4918 valid questionnaires. The mean age of the participants was 30.1 years (standard deviation = 12.5). The correlation analysis demonstrated a significant negative association between family health and suboptimal health status (r = −0.44; *P* < 0.001). The results of the parallel mediation analysis showed that family health had a significant indirect effect on suboptimal health status through both sleep quality (β = -0.350; *P* < 0.001) and health behaviour (β = −0.137; *P* < 0.001). The total indirect effect of family health on suboptimal health status through both sleep quality and health behaviour was also significant (β = −0.569, *P* < 0.001).

**Conclusions:**

This study highlights the significance of family health as a predictor of suboptimal health status and suggests that sleep quality and health behaviour are parallel mediators in this relationship. By understanding the role of family health, sleep quality, and health behaviour, interventions can be targeted to improve overall health outcomes.

Suboptimal health is a condition in which individuals encounter diverse health issues that do not meet the criteria for a specific disease diagnosis [1]. It is characterised by unexplained deterioration of physiological function that falls health and illness, and is marked by a variety of symptoms (e.g. fatigue and non-specific pain) which have a substantial impact on individuals’ overall well-being and daily functioning [2,3]. Despite not being classified as a disease, numerous studies have indicated that suboptimal health may precede the onset of chronic diseases [4,5], making it a notable public health concern.

Understanding the factors that contribute to suboptimal health is crucial for developing effective preventive and intervention strategies. In this sense, the significance of family health in influencing individuals' health outcomes has been increasingly acknowledged in recent years [6-8]. Conceptually, family health emerges from the convergence of the health status of each family member; their interactions and abilities; and the family's tangible, social, emotional, financial, and medical resources [9]. It involves a family’s social and emotional health processes, healthy lifestyle, health resources, and external social supports [10]. However, suboptimal health status of individuals is not only influenced by family health, but also by their sleep quality and health behaviour [11,12]. Sleep plays a vital role in the maintenance of good health, and poor sleep quality is associated with an increased risk of suboptimal health [11,12]. Furthermore, adopting healthy behaviours, such as maintaining a balanced diet and abstaining from tobacco and alcohol consumption, can enhance health outcomes and mitigate the likelihood of chronic diseases [13]. Conversely, engaging in unhealthy behaviours, such as consuming a poor diet and abusing substances, contributes to the development of suboptimal health [11,12].

Previous research has explored the separate connections between family health, sleep quality, and health behaviour with suboptimal health status. However, there is a lack of comprehensive understanding regarding their combined effects. Prior studies have demonstrated the mediating influences of health behaviour and sleep quality on family health and health outcomes [14]. In relation to this gap, we aimed to examine the simultaneous mediating role of sleep quality and health behaviour in the association between family health and suboptimal health status. We set two hypotheses: That there is a negative correlation between family health and suboptimal health status, and that both sleep quality and health behaviour play a parallel mediation role in the relationship between family health and suboptimal health status.

## METHODS

### Study design

We used a cross-sectional study design ad reported our findings per the STROBE reporting guidelines [15].

### Study setting and participants

Of the 31 provinces in China, we conducted this study in Guangdong Province and Jiangsu Province in the east; Sichuan Province in the west; and Macao Special Administrative Region, which rank ninth, fifth, twenty-second, and second in terms of GDP per capita in 2022, respectively [16]. We collected our data between 16 June 2023 to 23 September 2023 among individuals aged ≥18 years residing in the aforementioned four regions, who could understand, read, and write Chinese, and were willing to participate. We excluded individuals who were unable to independently complete the online questionnaire. Since there was limited information available, we assumed that the effect of family health on sleep quality and health behaviour and the effect of sleep quality and health behaviour on suboptimal health status would be small, resulting in maximum variability. Therefore, we used power tables which indicated that a sample size of 558 or more was necessary to achieve a power of 0.8 when employing percentile bootstrap for testing the mediating effect [17].

### Procedures

We recruited participants using a combination of non-probability sampling techniques – convenience sampling and respondent-driven sampling. Specifically, we reached out to leaders of higher education institutions, community service agencies, and large enterprises, requesting their collaboration in disseminating posters containing a QR code and a link to the survey among eligible participants within their organisations. Additionally, we used social media platforms such as WeChat, Facebook, Instagram, and WhatsApp to share the poster.The poster itself explicitly encouraged viewers to forward them to individuals who met the eligibility criteria. For the survey itself, we used Wenjuanxing, a widely recognised online survey platform in China. Each IP address and device could only submit the questionnaire once to ensure data quality and avoid duplicate responses.

### Measures

The questionnaire comprised of five sections. The first collected demographic information: gender, age, education, marital status, and employment status. The second section relating to family health included the Family Health Scale-Short Form (FHS-SF) developed by Crandall et al. [10]. This scale employs a five-point Likert scale ranging from 1 (strongly disagree) to 5 (strongly agree) and consists of ten items, three of which are reverse scored. The total score on the FHS-SF ranges from 10 to 50, with higher scores indicating a greater level of family health. The Cronbach’s α of the Chinese version of the FHS-SF used in this study was 0.83 [18]. The third section contained the Suboptimal Health Status Questionnaire (SHSQ-25), comprising 25 items with responses on a five-point Likert scale ranging 0 (never or almost never) to 4 (always), resulting in a score ranging from 0 to 100, with a score of 0 representing the lowest level of suboptimal health status (indicating good health) and a score of 100 representing the highest level (indicating poor health) [19]. A score of ≥5 is considered indicative of suboptimal health status, while scores below this threshold indicate ideal health [20]. We used the Chinese version of the SHSQ-25, with a Cronbach’s α of 0.93. The fourth part queried the participants’ sleep quality with the Self-Rating Scale of Sleep (SRSS), which comprises 10 items evaluated on a five-point Likert scale (Cronbach’s α = 0.64). The total score on this scale ranges from 10 to 50, with higher scores indicating lower sleep quality [21]. Finally, the fifth section looked at health behaviour using the Health Behaviour Inventory – Short Form (HBI-SF), which uses a seven-point Likert scale ranging from 1 (strongly disagree) to 7 (strongly agree) and comprises 12 items, with six items being reverse scored. It consists four subscales: The diet subscale (Cronbach’s α = 0.81), the proper use of health care resources subscale (Cronbach’s α = 0.81), the anger and stress subscale (Cronbach’s α = 0.62), and the substance use subscale (Cronbach's α = 0.73) [22]. The total score and subscale scores are derived by summing the participants’ responses and dividing by the number of items, and higher scores indicate a higher level of health risk.

### Data analysis

We summarised the participants’ characteristics through descriptive statistics and presented them through means and standard deviations (SDs) or frequencies and percentages. We performed correlation analyses to examine the associations among family health, suboptimal health status, sleep quality, and health behaviour, reporting the measures through the Pearson correlation coefficient and visualising them through heat maps. We also employed multiple linear regression models examine the combined effects of family health, sleep quality, and health behaviour on suboptimal health status, which we adjusted for potential confounding variables (gender, age, education, marital status, and employment status). To assess the potential mediation effects of sleep quality and health behaviour in the relationship between family health and suboptimal health status, we set up a structural equation model using maximum likelihood estimation. We evaluated its goodness-of-fit calculating the comparative fit index (CFI), goodness-of-fit index (GFI) and standardised root mean residual (SRMR), with acceptable thresholds set at >0.90, >0.90, and <0.10, respectively [23]. To further clarify the association between family health and suboptimal health, we conducted subgroup analyses at different educational levels, including secondary or below education and tertiary or higher education. Additionally, we performed the Sobel test as a sensitivity analysis to evaluate the robustness of the results. We performed all analyses in jamovi, 2.3.28 (jamovi team, Sydney, Australia), with the significance threshold set at *P* < 0.05.

## RESULTS

### Demographic characteristics

W collected 4918 valid questionnaires. The mean age of the participants was 30.1 years (SD = 12.5). Most were female (73.6%), had tertiary education (60.1%), were single (55.1%), and were unemployed (50.3%) (**Table 1**).

**Table 1:** Participant demographic data (n = 4918)*

Variable	
Gender, n (%)	
*Female*	3620 (73.6)
*Male*	1298 (26.4)
Age, mean (SD)	30.1 (12.6)
Highest educational attainment, n (%)	
*Secondary or below*	1685 (34.3)
*Tertiary*	2958 (60.1)
*Postgraduate*	275 (5.6)
Marital status, n (%)	
*Single*	2709 (55.1)
*Married*	2131 (43.3)
*Other (divorced or widow)*	78 (1.6)
Employment status, n (%)	
*Full-time employed*	2207 (44.9)
*Part-time employed*	238 (4.8)
*Unemployed (including retired, homemaker, and student)*	2473 (50.3)
Family health, mean (SD)	32.2 (5.7)
Suboptimal health status, mean (SD)	29.6 (17.9)
*Ideal health*	3165 (64.4)
*Suboptimal health status*	1753 (35.6)
Sleep quality, mean (SD)	20.8 (6.5)
Health behaviour, mean (SD)	3.1 (0.8)

SD – standard deviation

### The association between family health and suboptimal health status

**Figure 1:**
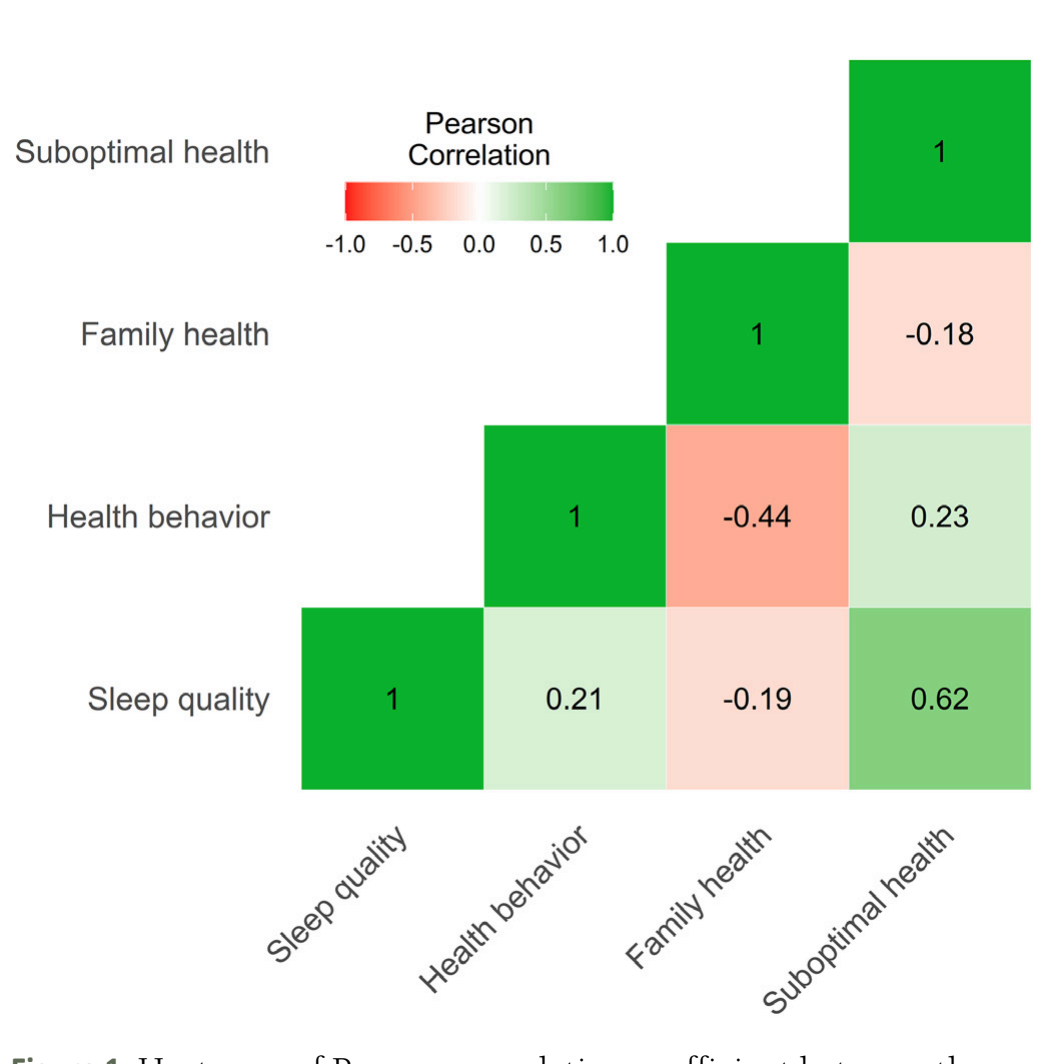
Heat map of Pearson correlation coefficient between the studied variables. Correlation significant at *P* < 0.001

The correlation analysis demonstrated a significant negative association between family health and suboptimal health status (r = −0.44; *P* < 0.001) (**Figure 1**). Moreover, 40.8% of the total variation of suboptimal health status can be explained by six independent variables (age, gender, employment status, family health, health behaviour, and sleep quality). After completely controlling for the other factors, the level of suboptimal health status decreased by 0.092 units when the family health level increased by one unit (β = −0.092; *P* < 0.018) (**Table 2**).

**Table 2:** Association between family health and suboptimal health status, explored by multiple linear regression*

Predictor	Estimate (95% CI)	SE	*t*	*P*-value
Intercept	−12.277 (−16.526, −8.027)	2.168	−5.664	<0.001
Age	0.107 (0.054, 0.159)	0.027	4.001	<0.001
*Health behaviour*	3.013 (2.416, 3.610)	0.305	9.893	<0.001
*Sleep quality*	1.613 (1.551, 1.675)	0.032	50.906	<0.001
*Family health*	−0.092 (−0.168, −0.015)	0.039	−2.359	0.018
Gender (females as reference)				
*Male*	−2.164 (−3.070, −1.258)	0.462	−4.682	<0.001
Highest educational attainment (secondary or below as reference)				
*Tertiary*	−0.181 (−1.074, 0.712)	0.455	−0.397	0.692
*Postgraduate*	0.582 (−1.271, 2.435)	0.945	0.616	0.538
Marital status (single as reference)				
*Married*	0.042 (−1.450, 1.534)	0.761	0.056	0.956
*Other*	−0.359 (−3.706, 2.987)	1.707	−0.211	0.833
Employment status (full-time employed as reference)				
*Unemployed*	−1.321 (−2.518, −0.123)	0.611	−2.162	0.031
*Part-time employed*	−1.15 (−3.045, 0.744)	0.966	−1.191	0.234

CI – confidence interval, SE – standard error

*R^2^ = 0.408.

### Mediation analysis

**Figure 2:**
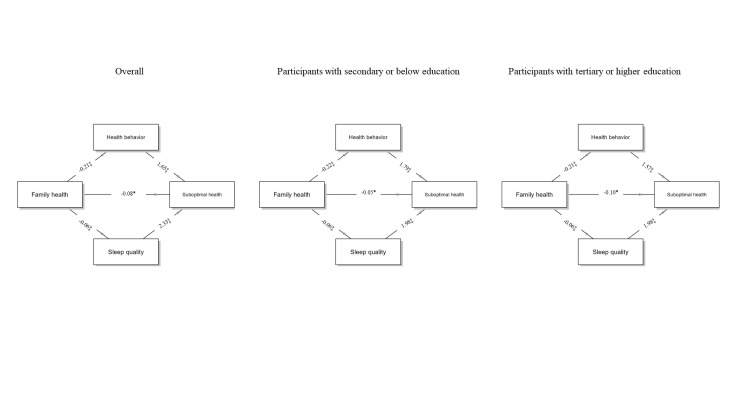
Parallel mediation model showing the effect of family health on suboptimal health. *P < 0.05. †*P* < 0.01. ‡*P* < 0.001

The direct effect analysis showed a negative correlation between family health and suboptimal health status (β = −0.082; *P* = 0.038). The results of the parallel mediation analysis demonstrated that family health had a significant indirect effect on suboptimal health status through both sleep quality (β = −0.350; *P* < 0.001) and health behaviour (β = −0.137; *P* < 0.001). Furthermore, the total indirect effect of family health on suboptimal health status through both sleep quality and health behaviour was also significant (β = −0.569; *P* < 0.001). The model fit statistics further confirmed the appropriateness of the model (CFI = 0.974, GFI = 0.999, SRMR = 0.031). The results of the subgroup analysis suggested that both the secondary or below education group and the tertiary or higher education group showed a significant parallel mediation role of sleep quality and health behaviour (**Figure 2**, **Table 3**).

**Table 3:** Summary of the results of the mediation analysis explored by maximum likelihood estimation*

Effect by type	Estimate (95% CI)	SE	*P*-value
Indirect			
*Family health → health behaviour → suboptimal health*	−0.137 (−0.172, (−0.102)	0.018	<0.001
*Family health → sleep quality → suboptimal health*	−0.35 (−0.403, −0.297)	0.027	<0.001
Component			
*Family health → health behaviour*	−0.059 (−0.062, −0.055)	0.002	<0 .001
*Health behaviour → suboptimal health*	2.335 (1.76, 2.91)	0.293	<0.001
*Family health → sleep quality*	−0.212 (−0.243, −0.181)	0.016	<0 .001
*Sleep quality → suboptimal health*	1.65 (1.588, 1.711)	0.031	<0 .001
Direct			
*Family health → suboptimal health*	−0.082 (−0.159, −0.005)	0.039	0.038
Total			
*Family health → suboptimal health*	−0.569 (−0.654, −0.483)	0.044	<0 .001

CI – confidence interval, SE – standard error

*Model fit indices are CFI = 0.974, GFI = 0.999, and SRMR = 0.031

### Sensitivity analysis

In view of the sensitivity analysis we performed to assess the robustness of the findings, the Sobel test showed that the primary results remained largely unchanged, suggesting a high level of stability in our findings (**Table 4**).

**Table 4:** Summary of the results of the mediation analysis, explored by Sobel test

Effect by type	Estimate (95% CI)	SE	*P*-value
Indirect			
*Family health → health behaviour → suboptimal health*	−0.137 (−0.172, −0.102)	0.018	<0.001
*Family health → sleep quality → suboptimal health*	−0.35 (−0.403, −0.297)	0.027	<0.001
Component			
*Family health → health behaviour*	−0.059 (−0.062, −0.055)	0.002	<0.001
*Health behaviour → suboptimal health*	2.335 (1.754, 2.916)	0.296	<0.001
*Family health → sleep quality*	−0.212 (−0.243, −0.181)	0.016	<0.001
*Sleep quality → suboptimal health*	1.65 (1.588, 1.711)	0.032	<0.001
Direct			
*Family health → suboptimal health*	−0.082 (−0.158, −0.005)	0.039	0.036
Total			
*Family health → suboptimal health*	−0.569 (−0.655, −0.483)	0.044	<0.001

CI – confidence interval, SE – standard error

## DISCUSSION

To our knowledge, this is the first study to assess the effect of family health on suboptimal health status, with the findings suggesting a negative correlation. Furthermore, both sleep quality and health behaviour were identified as mediators in this relationship. These results highlight the importance of considering family health, sleep quality, and health behaviour in understanding and addressing suboptimal health status.

Based on our findings, family health has a significant and inverse relationship with suboptimal health status, suggesting that individuals with higher levels of family health are less likely to experience suboptimal health status. There are several possible explanations for this relationship. First, a healthy family environment often promotes healthy lifestyle behaviours, such as regular physical activity and balanced nutrition [24], which are known to improve health outcomes and decrease the risk of developing suboptimal health status [11]. Second, a supportive and nurturing family environment can contribute to lower stress levels [25]. Notably, chronic stress has been associated with various negative health effects, including suboptimal health status [26]. In contrast, a strong support system within the family can mitigate the impact of stress and enhance resilience [27], leading to better health outcomes. Furthermore, family health can also influence the availability and access to health care resources. Families with better health may be more likely to prioritise health care and seek timely medical attention when needed [28]. This proactive approach to health management can prevent the development or progression of suboptimal health status. In general, family health, especially their healthy lifestyles and health resources, significantly affects proximal health processes that ultimately determine long-term health outcomes [7]. Our findings highlight the importance of considering the family context in understanding and promoting optimal health outcomes. Recognising the importance of family health in promoting optimal health outcomes can guide interventions and public health initiatives, while strategies aimed at improving family health, such as strengthening family support networks and providing resources for families in need, can potentially reduce the burden of suboptimal health in the population.

Our results also showed that sleep quality mediated the relationship between family health and suboptimal health status. Participants with poor family health tended to have lower sleep quality, which, in turn, was associated with a higher likelihood of experiencing suboptimal health status. These findings suggest that sleep quality may act as a mechanism through which family health influences individuals’ health status. Individuals with poor family health may experience higher levels of stress and emotional distress [25], which can negatively impact their sleep quality [29]. Moreover, family health encompasses various aspects such as physical health, mental well-being, and overall family functioning. Poor family health results in family members adopting sleep hygiene-harming behaviours, such as interfering with each other’s sleep patterns [30]. Family members may engage in behaviours such as talking or engaging in other activities that disrupt each other’s ability to fall or stay asleep. The detrimental impact of poor family health on the quality of sleep can subsequently result in a variety of adverse health outcomes. Previous studies have demonstrated that individuals who self-reported experiencing poor sleep quality consistently displayed elevated levels of physical health issues, such as compromised immune function [31] and an augmented susceptibility to chronic diseases [32]. Additionally, these participants consistently exhibited various adverse mental health indicators, such as increased fatigue [33] and stress [29]. Improving sleep quality may therefore serve as a potential pathway to enhance overall well-being and functioning in individuals with poor family health.

We also found that health behaviour mediated the relationship between family health and suboptimal health status. Specifically, individuals from healthier family environments tend to engage in healthier behaviours, such as balanced diets [34], not smoking [35], and limiting alcohol consumption [36], which in turn contributes to better health outcomes. However, individuals from less healthy family environments may be more prone to adopting unhealthy behaviours such as poor dietary habits, leading to suboptimal health status [11]. The mediating effect of health behaviour suggests that interventions targeting health behaviour may be effective in reducing the negative impact of poor family health on individual health outcomes. By promoting healthy behaviours such as a balanced diet and smoking cessation, individuals with poor family health can potentially improve their health status. Individuals with lower health literacy may struggle to comprehend and act upon health information, potentially hindering their ability to make positive health choices [37]. Therefore, efforts should be made to enhance health literacy among individuals and families, as this may enhance their ability to make informed decisions and engage in healthy behaviours [38]. Notably, the mediating effect of health behaviour does not negate the significance of family health, but rather highlights the potential pathways through which it influences individual health outcomes.

### Limitations

This study has some limitations. First, the cross-sectional design prevents us from making causal inferences about the relationship between family health, suboptimal health status, sleep quality, and health behaviours. Likewise, we only collected data at a single point in time; by following participants for a longer period, future studies could evaluate whether the observed changes in sleep quality and health-related behaviours are maintained or if they fade over time. Second, we measured all the variables of interest using self-report questionnaires, which are subject to recall bias and social desirability, meaning that participants may have underreported or overreported their responses, affecting the reliability and validity of our findings. Future research should consider employing objective measures such as physiological measures or observational techniques to enhance the accuracy and reliability of the data. Moreover, selection bias may exist in the participant sample, as we recruited participants through convenience sampling. Notably, our sample was predominantly female, with over 70% of participants being women. Additionally, more than 60% of our participants had tertiary education or higher. Finally, it is possible that individuals who were motivated to improve their health were more likely to participate in the study, which may have affected the generalisability of our findings. Future research should aim to address these limitations through long-term follow-up, objective measures, and a diverse participant sample.

## CONCLUSIONS

Our findings provide insights into the influence of family health on suboptimal health status and show a parallel mediation role of sleep quality and health behaviour in this relationship, highlighting the importance of considering these factors when assessing and addressing individual health outcomes. Further research should further explore the specific mechanisms through which family health influences sleep quality and health behaviour and should attempt to identify effective interventions to improve family health and enhance individual health.
